# Supplementary material for: Sorting at embryonic boundaries requires high heterotypic interfacial tension
Source: Nat Commun. 2017 Jul 31;8:157. doi: 10.1038/s41467-017-00146-x (PMC5537356; doi:10.1038/s41467-017-00146-x)
Supplement: Supplementary file 2 — Supplementary Software 1 [file 41467_2017_146_MOESM2_ESM.zip › PottsModel/SrcPottsModel/doc/model/IPositionManager.html]

IPositionManager


JavaScript is disabled on your browser.


Skip navigation links


- Overview
- Package
- Class
- Use
- Tree
- Deprecated
- Index
- Help

- Prev Class
- Next Class

- Frames
- No Frames

- All Classes

- Summary:
- Nested |
- Field |
- Constr |
- Method

- Detail:
- Field |
- Constr |
- Method


model

## Interface IPositionManager

- All Known Implementing Classes:
  :   HexagonPositionManager, SquarePositionManager

  ---

    

  ```
  public interface IPositionManager
  ```

- - ### Nested Class Summary

    Nested Classes

    | Modifier and Type | Interface and Description |
    | --- | --- |
    | `static class` | `IPositionManager.Position` |
  - ### Field Summary

    Fields

    | Modifier and Type | Field and Description |
    | --- | --- |
    | `static IPositionManager.Position[]` | `HexagonPositions` |
    | `static IPositionManager.Position[]` | `SquarePositions` |
  - ### Method Summary

    All Methods Instance Methods Abstract Methods

    | Modifier and Type | Method and Description |
    | --- | --- |
    | `boolean` | `areHorizontalNeighbours(Pixel pPixel1, Pixel pPixel2)` |
    | `boolean` | `areNeighbours(Pixel pPixel1, Pixel pPixel2)` |
    | `boolean` | `areVerticalNeighbours(Pixel pPixel1, Pixel pPixel2)` |
    | `IPositionManager.Position[]` | `getAllNeighborPositions()` Defines neighbor positions for given position model. |
    | `Pixel[]` | `getAllNeighbors(Pixel pPixel)` Returns an array of APixels whose length corresponds to the number of possible positions. |
    | `Pixel[]` | `getAllNeighbors(Pixel pPixel1, Pixel pPixel2)` Get union of neighbors of pPixel1 and pPixel2. |
    | `IPositionManager.Position[]` | `getConnectedNeighborPositions()` |
    | `Pixel[]` | `getConnectedNeighbors(Pixel pPixel)` |
    | `Pixel` | `getNeighborFromPosition(Pixel pPixel, IPositionManager.Position pPosition)` |
    | `IPositionManager.Position[]` | `getPartialNeighborPositions()` Returns partial neighbors so that iterating over all pixels and getting their respective partial neighbors will only result in passing over them a single time. |
    | `Pixel[]` | `getPartialNeighbors(Pixel pPixel)` |

- - ### Field Detail


    - #### SquarePositions

      ```
      static final IPositionManager.Position[] SquarePositions
      ```


    - #### HexagonPositions

      ```
      static final IPositionManager.Position[] HexagonPositions
      ```
  - ### Method Detail


    - #### getAllNeighbors

      ```
      Pixel[] getAllNeighbors(Pixel pPixel)
      ```

      Returns an array of APixels whose length corresponds to the number of possible positions.
      However, some entries may be Null!

      Parameters:
      :   `pPixel` -

      Returns:


    - #### getConnectedNeighbors

      ```
      Pixel[] getConnectedNeighbors(Pixel pPixel)
      ```


    - #### getAllNeighbors

      ```
      Pixel[] getAllNeighbors(Pixel pPixel1,
                              Pixel pPixel2)
      ```

      Get union of neighbors of pPixel1 and pPixel2.

      Parameters:
      :   `pPixel1` -
      :   `pPixel2` -

      Returns:


    - #### getPartialNeighbors

      ```
      Pixel[] getPartialNeighbors(Pixel pPixel)
      ```


    - #### getAllNeighborPositions

      ```
      IPositionManager.Position[] getAllNeighborPositions()
      ```

      Defines neighbor positions for given position model.

      Returns:


    - #### getPartialNeighborPositions

      ```
      IPositionManager.Position[] getPartialNeighborPositions()
      ```

      Returns partial neighbors so that iterating over all pixels and getting their respective
      partial neighbors will only result in passing over them a single time. This is useful
      when computing the heterotypic boundary length for example and avoiding counting edges twice.

      Returns:


    - #### getConnectedNeighborPositions

      ```
      IPositionManager.Position[] getConnectedNeighborPositions()
      ```


    - #### getNeighborFromPosition

      ```
      Pixel getNeighborFromPosition(Pixel pPixel,
                                    IPositionManager.Position pPosition)
      ```

      Parameters:
      :   `pPixel` -
      :   `pPosition` -

      Returns:
      :   pPixel's neighbor from position pPosition or Null if the neighbor is out of bounds


    - #### areNeighbours

      ```
      boolean areNeighbours(Pixel pPixel1,
                            Pixel pPixel2)
      ```


    - #### areHorizontalNeighbours

      ```
      boolean areHorizontalNeighbours(Pixel pPixel1,
                                      Pixel pPixel2)
      ```


    - #### areVerticalNeighbours

      ```
      boolean areVerticalNeighbours(Pixel pPixel1,
                                    Pixel pPixel2)
      ```


Skip navigation links


- Overview
- Package
- Class
- Use
- Tree
- Deprecated
- Index
- Help

- Prev Class
- Next Class

- Frames
- No Frames

- All Classes

- Summary:
- Nested |
- Field |
- Constr |
- Method

- Detail:
- Field |
- Constr |
- Method
